# Supplementary material for: Prevalence and predictors of prenatal depression during the COVID-19 pandemic: A multistage observational study in Beijing, China
Source: PLoS One. 2024 Apr 25;19(4):e0298314. doi: 10.1371/journal.pone.0298314 (PMC11045078; doi:10.1371/journal.pone.0298314)
Supplement: S1 Table — (DOCX) [file pone.0298314.s001.docx]

**Homogeneity test of confounders**

Aiming to analyze the prevalence difference among the 3 phases of the COVID-19 pandemic, we first conducted a Pearson Chi-square or Fisher’s exact test (while appropriate) to examine the confounding bias. Table S1 showed all demographic, pregnancy-related, and psychosocial characteristics among phases of COVID-19 pandemic were homogenous.

**S1** **Table. Homogeneity test of** **confounders among different phases of COVID-19 in Beijing, China**^a^

| Variables | Pre-COVID-19  n (%) | Peak-COVID-19  n (%) | Post-COVID-19 n (%) | χ^2 b^ | *P* |
| --- | --- | --- | --- | --- | --- |
| age (years) |  |  |  | 8.220 | 0.211 |
| 20-25 | 5(55.6) | 1(11.1) | 3(33.3) |  |  |
| 26–30 | 81(38.0) | 61(28.6) | 71(33.3) |  |  |
| 31–35 | 160(42.8) | 73(19.5) | 141(37.7) |  |  |
| >35 | 64(43.8) | 36(24.7) | 46(31.5) |  |  |
| BMI^c^ |  |  |  | 8.287 | 0.242 |
| underweight | 18(36.7) | 12(24.5) | 19(38.8) |  |  |
| normal weight | 222(43.9) | 115(22.7) | 169(33.4) |  |  |
| overweight | 61(39.4) | 39(25.2) | 55(35.5) |  |  |
| obese | 9(28.1) | 5(15.6) | 18(56.3) |  |  |
| education attainment |  |  |  | 2.963 | 0.564 |
| college degree or below | 24(38.1) | 19(30.2) | 20(31.7) |  |  |
| bachelor degree | 133(42.0) | 76(24.0) | 108(34.1) |  |  |
| master degree or above | 153(42.3) | 76(21.0) | 133(36.7) |  |  |
| occupation |  |  |  | 1.665 | 0.435 |
| employed | 296(41.6) | 167(23.5) | 249(35.0) |  |  |
| unemployed | 14(46.7) | 4(13.3) | 12(40.0) |  |  |
| history of mental illness^d^ |  |  |  | 0.356 | 0.933 |
| yes | 5(41.7) | 2(16.7) | 5(41.7) |  |  |
| no | 305(41.8) | 169(23.2) | 256(35.1) |  |  |
| co-living |  |  |  | 7.262 | 0.297 |
| husband only | 164(40.8) | 94(23.4) | 144(35.8) |  |  |
| alone | 18(34.0) | 13(24.5) | 22(41.5) |  |  |
| Husband’s family | 59(44.0) | 37(27.6) | 38(28.4) |  |  |
| others | 69(45.1) | 27(17.6) | 57(37.3) |  |  |
| per capita monthly household income^e^ |  |  |  | 3.617 | 0.164 |
| non-poverty | 292(42.7) | 153(22.4) | 239(34.9) |  |  |
| poverty | 18(31.0) | 18(31.0) | 22(37.9) |  |  |
| per capita living space^f^ |  |  |  | 3.505 | 0.173 |
| non-small | 218(43.8) | 106(21.3) | 174(34.9) |  |  |
| small | 92(37.7) | 65(26.6) | 87(35.7) |  |  |
| gestational weeks^g^ |  |  |  | 2.912 | 0.573 |
| first | 74(41.3) | 36(20.1) | 69(38.5) |  |  |
| second | 168(40.7) | 99(24.0) | 146(35.4) |  |  |
| third | 68(45.3) | 36(24.0) | 46(30.7) |  |  |
| gravidity ^h^ |  |  |  | 0.344 | 0.842 |
| primi-gravida | 148(40.8) | 84(23.1) | 131(36.1) |  |  |
| multi-gravida | 162(42.7) | 87(23.0) | 130(34.3) |  |  |
| history of abortion^i^ |  |  |  | 0.377 | 0.828 |
| yes | 42(39.6) | 24(22.6) | 40(37.7) |  |  |
| no | 268(42.1) | 147(23.1) | 221(34.7) |  |  |
| pregnancy intentions |  |  |  | 3.791 | 0.150 |
| planned conception | 197(40.3) | 108(22.1) | 184(37.6) |  |  |
| unplanned pregnancy | 113(44.7) | 63(24.9) | 77(30.4) |  |  |
| perception of family care |  |  |  | 6.639 | 0.156 |
| satisfied | 278(41.2) | 157(23.3) | 240(35.6) |  |  |
| medium | 20(51.3) | 11(28.2) | 8(20.5) |  |  |
| dissatisfied | 12(42.9) | 3(10.7) | 13(46.4) |  |  |
| expected mode of delivery |  |  |  | 4.239 | 0.375 |
| vaginal delivery | 180(40.2) | 106(23.7) | 162(36.2) |  |  |
| cesarean delivery | 40(47.6) | 22(26.2) | 22(26.2) |  |  |
| not considered | 90(42.9) | 43(20.5) | 77(36.7) |  |  |
| boy-preference^j^ |  |  |  | 5.139 | 0.148 |
| 0 | 268(41.1) | 147(22.5) | 237(36.3) |  |  |
| 1 | 36(50.0) | 18(25.0) | 18(25.0) |  |  |
| 2 | 6(33.3) | 6(33.3) | 6(33.3) |  |  |
| social support |  |  |  | 5.169 | 0.075 |
| high | 176(39.6) | 98(22.0) | 171(38.4) |  |  |
| low | 134(45.1) | 73(24.6) | 90(30.3) |  |  |

Abbreviation: COVID-19, coronavirus disease 2019.

^a^Pre-COVID-19 (March 28, 2019-November 01, 2019), Peak-COVID-19 (January 20, 2020- August 25, 2020), and Post-COVID-19 (October 01, 2020-May 07, 2021).

^b^Fisher’s exact test was conducted when any expectations < 5.

^c^Calculated as weight in kilograms divided by height in meters squared; stratified as underweight (<18.5 kg/m^2^), normal weight (18.5–23.9 kg/m^2^), overweight (24–27.9 kg/m^2^), and obese (≥28 kg/m^2^).

^d^Had a history of a diagnosed mental disorder prior to pregnancy.

^e^Calculation formula was the average monthly household income in the previous year divided by the number of adults and children. 5000 RMB was defined as the poverty line according to data from the Beijing statistical yearbook, Beijing Bureau of Statistics.

^f^Defined as family living space divided by the number of adults and children and 21 m^2^ was signed as a cut-off point of small size.

^g^Categorized as the first (≤12 weeks of gestation), second (13–26 weeks of gestation), and third (＞26 weeks of gestation) trimester.

^h^Primi-gravida (first pregnancy) and multi-gravida (≥2 pregnancy experiences).

^i^Covered both induced and spontaneous abortion.

^j^Categorized by the number of boy-preference for husband’s family and pregnant women.
