# Supplementary material for: Prevalence and predictors of prenatal depression during the COVID-19 pandemic: A multistage observational study in Beijing, China
Source: PLoS One. 2024 Apr 25;19(4):e0298314. doi: 10.1371/journal.pone.0298314 (PMC11045078; doi:10.1371/journal.pone.0298314)
Supplement: Attachment — Submitted filename: Response to Reviewers.docx [file pone.0298314.s003.docx]

Dear Editor and Reviewers:

Thank you for your letter and for the reviewers’ comments concerning our manuscript entitled “Prevalence and Predictors of Prenatal Depression during the COVID-19 Pandemic: A Multistage Observational Study in Beijing, China” (ID: PONE-D-22-21013). Those comments are valuable and very helpful for revising and improving our paper, as well as the important guiding significance to our research. We have studied comments carefully and have made correction which we hope meet with approval. Your suggestions and comments are italicized and our response is given in red.

**Suggestion and Response to academic editor**

*1. Please ensure that your manuscript meets PLOS ONE's style requirements, including those for file naming.* *The PLOS ONE style templates can be found at https://journals.plos.org/plosone/s/file?id=wjVg/PLOSOne_formatting_sample_main_body.pdf and https://journals.plos.org/plosone/s/file?id=ba62/PLOSOne_formatting_sample_title_authors_affiliations.pdf.*

We have carefully studied the two documents mentioned above and revised our paper in accordance with the PLOS ONE style templates, including title, authors, affiliations, and main body. Please feel free to contact us if anything improper exists.

*2. Thank you for stating in your Funding Statement:*

*“This research received support from the Capital Clinical Application Research Founding of Beijing Municipal Science and Technology Committee (Grant number: Z181100001718031). The funder did not involve in any part of the study process, from design to submit the article for publication.”*

*Please provide an amended statement that declares *all* the funding or sources of support (whether external or internal to your organization) received during this study, as detailed online in our guide for authors at http://journals.plos.org/plosone/s/submit-now. Please also include the statement “There was no additional external funding received for this study.” in your updated Funding Statement.*

*Please include your amended Funding Statement within your cover letter. We will change the online submission form on your behalf.*

Funding Statement has been updated as your suggestion and attached at the end of the cover letter 2.

*3. PLOS requires an ORCID iD for the corresponding author in Editorial Manager on papers submitted after December 6th, 2016. Please ensure that you have an ORCID iD and that it is validated in Editorial Manager. To do this, go to ‘Update my Information’ (in the upper left-hand corner of the main menu), and click on the Fetch/Validate link next to the ORCID field. This will take you to the ORCID site and allow you to create a new iD or authenticate a pre-existing iD in Editorial Manager. Please see the following video for instructions on linking an ORCID iD to your Editorial Manager account:* [*https://www.youtube.com/watch?v=_xcclfuvtxQ*](https://www.youtube.com/watch?v=_xcclfuvtxQ)*.*

We have authenticated a pre-existing ORCID iD (0000-0002-3527-4859) in Editorial Manager.

*4. Thank you for stating the following in the Acknowledgments Section of your manuscript:*

*“This research received support from the Capital Clinical Application Research Founding of Beijing Municipal Science and Technology Committee (Grant number: Z181100001718031). The funder did not involve in any part of the study process, from design to submit the article for publication.”*

*We note that you have provided additional information within the Acknowledgements Section that is not currently declared in your Funding Statement. Please note that funding information should not appear in the Acknowledgments section or other areas of your manuscript. We will only publish funding information present in the Funding Statement section of the online submission form.*

*Please remove any funding-related text from the manuscript and let us know how you would like to update your Funding Statement. Currently, your Funding Statement reads as follows:*

*“This research received support from the Capital Clinical Application Research Founding of Beijing Municipal Science and Technology Committee (Grant number: Z181100001718031). The funder did not involve in any part of the study process, from design to submit the article for publication.”*

*Please include your amended statements within your cover letter; we will change the online submission form on your behalf.*

Funding Statement has been amended and attached at the end of cover letter 2. Funding information in the manuscript has been removed.

**Response to the Reviewer’s comments:**

**Reviewer #1:**

*1. My first concern is about the risk of bias in collecting data and reporting results in this study. Authors measured depression (interviews/survey) in different individuals in different phases of Covid 19 outbreak. We are aware that depression depends on various factors like socio-demographic characteristics, economic status, age, number of pregnancies, and mental status at the time of the study (as the authors acknowledged and discussed in the method section). However, I am still uncertain how authors reduced the risk of bias in reporting the prevalence in different phases of Covid 19 outbreak. I mean, how did they relate a higher rate of depression in certain individuals who were surveyed during the peak phase to the outbreak? For example, those who attended the study during the peak phase are not the same individuals who did so before or post the peak phase. I like to see what strategies the authors applied to reduce the risk of bias in reporting results.*
It is really true as Reviewer suggested that the reduction of bias risk is very important for a multi-stage observational study, we have made an addition in Strengths and limitations section to illustrate it (Page 20, line 411-416 in “Revised Manuscript with Track Changes”).

Given that prenatal depression can only be measured during pregnancy and the unpredictability of COVID-19 development trajectory, longitudinal studies are almost unrealistic. This means that we were unable to recruit the same samples to conduct this study. Therefore, we assessed the homogeneity of confounding variables across three phases, such as age, BMI, education attainment, etc. Fortunately, Table S1 indicated that demographic, pregnancy-related, and psychosocial characteristics across three phases were homogenous. For this reason, we inference that a significant variation in the prevalence of prenatal depression may be partially due to the different phases of the COVID-19.

*2. Another concern is about collecting data for the pre-peak phase of covid 19. How did the authors collect data? Did you conduct the study from March 2019 to May 2021, then classified the collected data into 3 phases (after completing data collection)?! Is that right? I mean, how did the authors predict there would be a peak phase for Covid 19? I would like to see an explanation about this.*

We are very grateful for your concern. Our hospital was appointed as the prenatal psychological care service at November, 2018 and since then we started to collect data. Considering the duration of the peak-COVID-19, equal days, and elimination of residual effects, we intercepted data from March 28, 2019 to May 07, 2021.

**Reviewer #2:**

*1. The body of knowledge that your study added*

*What was the necessity for your research, given that there have already been studies on this topic in many regions of China that have taken into account essential factors that you overlooked, and did you feel that your research contributed anything new to the body of knowledge? (I have attached some of similar works conducted previously).*

*I noticed that there is a duplication of efforts.*

*• https://www.frontiersin.org/articles/10.3389/fpsyt.2023.1191152/full*

*• https://bmcpregnancychildbirth.biomedcentral.com/articles/10.1186/s12884-022-04428-1*

*•* [*https://bmjopen.bmj.com/content/10/9/e038511*](https://bmjopen.bmj.com/content/10/9/e038511)

After carefully study of literatures listed above, we have re-written introduction (Page 4, line 91-96 and page 5, line 97-106 in “Revised Manuscript with Track Changes”) and discussion (Page 18, line 355-359 in “Revised Manuscript with Track Changes”) section according to the Reviewer’s suggestion.

Firstly, the duration of these studies’ investigation was relatively short, focusing on a particular phase of the COVID-19 separately, and unable to provide a comprehensive picture of the prevalence of prenatal depression throughout the epidemic in China. By contrast, our research was a multistage observational study, and revealed the impact of the COVID-19 on prenatal depression by comparing the prevalence of prenatal depression among pre-, peak-, and post-epidemic. In addition, none of them involved gender preference in the analysis of relevant factors, which is a significant variable for prenatal depression in low- and middle-income countries, and this is exactly one highlight of our study. Therefore, we believe that this study is necessary and contributes something new.

*Moreover, the data was taken from 2019-2021, and it would have been interesting if it was accessible for the scientific community during the rapid raise of the covid-19 pandemic. However, it is too late to be used by the scientific community (two years later than the time of data collection).*

Just as Reviewer suggested, two years after data collection does appear to be a bit late for a study to be published. Be honest, we submitted our manuscript in June 2022, when the epidemic was still strictly controlled in Beijing. Despite the editor’s great efforts, it took nearly one year before the decision. We will do our best to make this research available to the scientific community as soon as possible.

*2. Abstract*

*The abstract did not adhere to the PLOS ONE’s manuscript submission guidelines:*

*The abstract should describe the main objective(s) of the study, the methods, the results and the conclusion. The subheading “Purpose” is not included in the PLOS ONE Manuscript Submission Guidelines.*

Thanks for your careful checks, we are very sorry for our incorrect writing. We have replaced the subheading “purpose” with “objective” according to submission guidelines (Page 1, line 12 in “Revised Manuscript with Track Changes”).

*3. Introduction*

*You stated ‘’Similarly, relatively high rates of symptoms of depression varying from 14.6% to 48.3% are reported in China, Spain, Italy, Iran, the US, Turkey, Nepal, and Denmark during the outbreak [8)’’. However, you missed the citation of all the references for studies conducted in various countries which actually indicated poor referencing for your evidences.*

We are appreciative of the carefulness of the reviewers. The literature we cited is a systematic review, and the original article stated that “**relatively high rates of symptoms of anxiety (6.33% to 50.9%), depression (14.6% to 48.3%), posttraumatic stress disorder (7% to 53.8%), psychological distress (34.43% to 38%), and stress (8.1% to 81.9%) are reported in the general population during the COVID-19 pandemic in China, Spain, Italy, Iran, the US, Turkey, Nepal, and Denmark”**. Therefore, we have not cited references for studies in various countries, please feel free to contact us if it is necessary.

*The gaps in the research published so far have not been adequately explained, nor the significance of your study compared to previous studies. The introduction also lacks focus to the study target (prenatal depression and its predicators). Moreover, the magnitude and important predictors of prenatal depression during the pandemic are not clearly stated. Overall, it needs general improvement.*

As Reviewer suggested that several potential predictors and COVID-19-related variables were important and not involved in our study. We have indicated this limitation in the “Strengths and limitations” section (Page 22, line 407-411 in “Revised Manuscript with Track Changes”). Furthermore, we have cited several similar studies in Introduction section, and explained the gaps in these studies to demonstrate the significance and implication of our work (Page 4, line 91-96 and page 5, line 97-102 in “Revised Manuscript with Track Changes”).

*4. Methods*

* Ethical issues*

*The eligibility criterion lacks clarity.*

*Inclusion criteria for eligible were pregnant women (2-9 months of pregnancy) who were aged 20 years and older, Chinese-speaking, confirmed their written consent, and had no plans to leave residence within 1 year. Women who had life-threatening illness or severe psychiatric disorders (e.g., bipolar disorder, schizophrenia or neurodevelopmental disorder) were excluded.*

*• Questions*

*Is it ethical to exclude non -Chinese speaking pregnant mothers, and planned to leave before one year but suffering from severe prenatal depression? What about those mothers with severe depression. Because, you stated that “Women with a high probability of prenatal depression were directed to contact free professional healthcare. How did you see the deliberate exclusion of mothers with depression from health care service based on their language from the ethical perspective? Why you included mothers >20years of age. What if mothers at the age of 18, 19?*

It is really true as Reviewer suggested that it is not ethical to exclude pregnant women who are non -Chinese speaking or plan to leave within 1 year. The reason for setting such inclusion criteria is that pregnant women suffering from prenatal depression in this study will be enrolled in a six-mouth psychological intervention, named “couple communication program”. Fortunately, no pregnant women were excluded due to these two criteria. We have removed these in accordance with reviewer’s suggestion (Page 6, line 121-122 in “Revised Manuscript with Track Changes”).

Mothers <20 years were excluded in the study to reduce confusion, since it is the legal age of marriage in China. Naturally, we will provide them with medical care as well.

* Assessment tool*

*• Psychological assessment tool*

*You stated “Psychosocial characteristics comprised mental history and social support’’. Here, the word mental history is a vague and stigmatizing word, it is better to replace with’ history of mental illness”*

Thank you for pointing this out. We have replaced the term “mental history” in the entire manuscript with “history of mental illness”.

*Based on a previous study, total scores were defined as low (< 44) and high (45-66). What if the score becomes exactly 44???*

We sincerely thank you for careful reading. After re-reading of the reference and confirmation by all authors, low social support was defined as ≤44 and we followed this definition in data analysis as well. We are very sorry for our mis-writing and have corrected in the manuscript (Page 9, line 202 in “Revised Manuscript with Track Changes”).

* Statistical analysis*

*• This sentence is ambiguous for the reader.*

*All data were categorized variables and were presented as absolute number and percentage (%). To identify the association between prevalence and phases of COVID-19, Pearson’s Chi-square was used after the examination of confounder’s homogeneity.*

We have revised this sentence for better understanding by the reader (Page 9, line 205-210 in “Revised Manuscript with Track Changes”).

*All variables with a p-value of 0.2 or lower in bivariate analysis were selected as potential predictors for the multivariate logistic regression model according to the suggestion of Hosmer and Lemeshow [37], crude odds ratios (cORs) and 95% confidence intervals (CIs) were calculated.*

*• Question*

*Why only variables with a p-value <=0.2 in the bivariate analysis were taken in to multi-variate analysis. It is generally recommended to included variables known to affect the dependent variable (prenatal depression in your case) in various studies rather than taking variables with a p-value<=0.2 in the bivariable analysis.*

Reviewer’s suggestion for variable inclusion is reasonable. For the same purpose, our selection for using a significance level as high as 0.2 as a screening criterion in the bivariable analysis is to prevent missing variables known to be important, just as recommendation by Hosmer and Lemeshow in **Applied Logistic Regression, “Our recommendation for using a significance level as high as 0.20 or 0.25 as a screening criterion for initial variable selection is based on the work by Bendel and Afif (1977) on linear regression and on the work by Mickey and Greenland (1989) on logistic regression. These authors show that use of a more traditional level (such as 0.05) often fails to identify variables known to be important.”**

Studies listed below adopted the same methodology.

<https://journals.plos.org/plosone/article?id=10.1371/journal.pone.0267189>

<https://thejns.org/search?q1=Development+of+a+prediction+rule+for+diagnosing+postoperative+meningitis%3A+a+cross-sectional+study>

https://www.dovepress.com/virological-treatment-failure-among-adult-hivaids-patients-from-select-peer-reviewed-fulltext-article-IDR

*• Operational definition*

*History of mental illness was not clearly operationalized and in Table 1 you defined mental history as “b Present depression, anxiety or psychosomatic symptoms prior to pregnancy” which is to mean history of common mental disorder. What about mothers with history of other mental health problems such as schizophrenia, bipolar etc.?. Did you include or not??*

We are very sorry for the ambiguous description of the “history of mental illness”. To be precise, “history of mental illness” means a history of a diagnosed mental disorder, whether common or severe mental illness. Mothers with a history of mental illness would not be excluded in this study. However, for the prevention of triggering symptoms, pregnant women undergoing severe mental disorder were excluded, but this does not mean we refuse to provide them with medical care. We have revised them in the manuscript (Page 6, line 122 and page 13, line 246-247 in “Revised Manuscript with Track Changes”) and supplementary material.

*5. Results*

*A total of 977 pregnant women received ANC from March 28, 2019 to May 07, 2021. Among them, 187 (19.1%) who rejected consent were excluded and 48 (4.9%) with missing data were deleted. Here, those who refuse to take consent should be counted as non-response rate and the response rate should be clearly stated.*

Considering Reviewer’s suggestion, we have added response rate (75.9%) to the manuscript (Page 10, line 231 in “Revised Manuscript with Track Changes”).

*Table 1*

*• It is better to replace “Number” with “Frequency”*

*• It is not self-explanatory; it did not show when, where the research is conducted as stated “Table 1 characteristic of the study participants”*

*• Figure 1 is difficulty to understand that needs clarity for the reader.*

*• It is generally replace bivariate with bivariable and multivariate with “multi variable”.*

We would like to thank you for your critical and constructive comments. According to your suggestion, we have revised in the following:

“Number” in Table 1 has been replaced with “Frequency” (Page 11 in “Revised Manuscript with Track Changes”).

Table 1 title has been mortified as “Characteristics of pregnant women throughout the COVID-19 in Beijing, China” (Page 10, line 241-242 in “Revised Manuscript with Track Changes”).

We have tried our best to clarify Figure 1 in the Result section. In addition, we have noted the figure as “The numbers in the blue and red bars represented the number of pregnant women without and with depression, and the percentages were prevalence of prenatal depression at each phase”. If anything absent or inappropriate, we really appreciate your help and would like very much to modify them.

“Bivariate” has been replaced with “bivariable” and “multivariate” with “multivariable” in the entire manuscript.

*Model fitness*

*The result of the Hosmer-Lemeshow test (P=0.034) indicated an acceptable fit of our multivariate regression model. However, it is true that a significant test indicates that the model is not a good fit and a non-significant test indicates a good fit. In this case the Hosmer-Lemeshow test is found to be significant which indicated the data is not explained by the model.*

We feel sorry for our carelessness. After re-analysis of the original data and repetitive checking, we realized that we had misplaced the zero after decimal point, which was a careless and major writing mistake. We are very grateful for your carefulness and have corrected 0.034 to 0.340 (Page 16, line 303 in “Revised Manuscript with Track Changes”).

*Predictor variables*

*You tried to assess factors that `potential affect the dependent variable. However, I noticed that important variables such as perceived stress, stressful life events, perceived stigma, substance history and history of medical illness among the participants and their families have been missed which at least would have been stated in the limitation part.*

We are very sorry for the weakness of the study design and will carefully consider these important variables in the future work. As Reviewer’s suggestion, we have revised the Strengths and limitations section, stating the absence of perceived stress, stressful life events, and substance history (Page 22, line 407-411 in “Revised Manuscript with Track Changes”).

*6. Discussion*

*Your discussion focused the comparison of your study with the previous studies but you did not address the clinical implication of your study findings. The direction of your recommendation for the scientific community is not clear.*

We are very grateful for your critical and constructive comments. We have re-written the Conclusion section according to your suggestion, including clinical implication of our results and recommendation for future studies (Page 20, line 426-430 and page 23, line 431-436 in “Revised Manuscript with Track Changes”).

Thank you again for your positive comments and valuable suggestions to improve the quality of our manuscript. If there are any other modifications required, we would appreciate your help and will try our best to revise them.

Sincerely,

Lihua LIU, PhD

Institution of Hospital Management, Medical Innovation Research Division of Chinese PLA General Hospital, Fuxing Road 28, 100853, Beijing, China
Email: qjldln888@163.com
