# Supplementary material for: Prevalence and predictors of prenatal depression during the COVID-19 pandemic: A multistage observational study in Beijing, China
Source: PLoS One. 2024 Apr 25;19(4):e0298314. doi: 10.1371/journal.pone.0298314 (PMC11045078; doi:10.1371/journal.pone.0298314)
Supplement: Attachment — Submitted filename: Response to Reviewers1.docx [file pone.0298314.s004.docx]

January 19, 2024

Dear Editor and Reviewers:

On behalf of my co-authors, I would like to express our gratitude for your opportunity to our manuscript titled “Prevalence and predictors of prenatal depression during the COVID-19 pandemic: a multistage observational study in Beijing, China” (ID: PONE-D-22-21013R3). We are very grateful for your constructive suggestions. Please accept our apologies for any inconvenience caused by our mistakes.

**After thoroughly reviewing and learning your suggestions, we have made the following changes to our submission:**

1. We have responded to each point raised by the reviewers in this letter.

2. We have uploaded two versions of our updated paper, one with track changes named “Revised Manuscript with Track Changes 4” and another unmarked version named “Manuscript 4” to the File Inventory.

3. As recommended by Reviewer #4, we have amended some abbreviations of the journals’ names.

4. We have replaced Figure 2 and Figure 3 with the re-rendered ones to improve their quality.

**Listed below are details of our point-to-point responses to the Academic Editor and reviewers to address all concerns. Your suggestions and comments were presented in italics and our responses were in red.**

**Comments and suggestions from the Reviewers and our responses**

**Reviewer #3:**

*The concerns I raised have been well addressed. Thank you and I wish you the best in the next phase of this manuscript submission.*

We are impressed by your friendly and positive comments throughout the review process, please accept our sincerest thanks and best wishes.

**Reviewer #4:**

*Table 1, line 250: All variables such as age, education attainment should begin with capital letters.*

We have amended them in Table 1 and thank you very much for your suggestion (Page 11, line 250 in “Revised Manuscript with Track Changes 4”).

*Line 296 (p values>0.05) should be presented as p>0.05.*

We have amended it and thank you very much for your suggestion (Page 15, line 296 in “Revised Manuscript with Track Changes 4”).

*Conclusion (line 425) "We concluded that the public and professionals should take prenatal depression seriously during the COVID-19" should read "We concluded that the public and professionals should take prenatal depression seriously during the COVID-19"*

We are very sorry for the confusion in your description. It seems that your statement does not differ from our paper. Please do not hesitate to point out the inappropriateness of our sentence clearly.

*Reference 25, the journal title "FRONT PSYCHIATRY" should capitalize first letters of the word as "Front Psychiatry".*

*Similarly line 559 reference 32 "LANCET GLOBAL HEALTH" should be written as "Lancet Global Health".*

*Line 693 reference 71 "MULTIDISCIPLINARY HEALTH" to be written as "Multidisciplinary Health".*

We are very impressed by your careful review. We have modified the abbreviations of the journals’ names as per your recommendation and will keep this in mind in future writing.

*The image quality of figure 2 and figure 3 is not very clear to enable reading the data output easily. Consider rendering these images (figures) afresh to improve on their quality.*

We have rendered and uploaded them. We are impressed by your rigorous research style.

Thank you for taking the time to provide us with guidance and suggestions, we have considered them to improve the quality of our manuscript with the hope of acceptance by PLOS ONE. Thank you for your patience and understanding throughout the process.

If you feel any further modifications are necessary, please do not hesitate to let us know; we will do our best to revise the manuscript accordingly. We are looking forward to hearing from you.

Thank you and best regards.

Sincerely,

Lihua LIU, PhD

Institution of Hospital Management, Medical Innovation Research Division of Chinese PLA General Hospital, Fuxing Road 28, 100853, Beijing, China
Email: qjldln888@163.com
